# A Novel Tertiary Carbamate Prodrug Strategy to Overcome Metabolic Barriers in Oral Ketamine Delivery

**DOI:** 10.1002/cmdc.202500856

**Published:** 2026-02-03

**Authors:** Juulia Järvinen, Santosh Kumar Adla, Janne Tampio, Aaro Jalkanen, Kenneth B. Sloan, Kristiina M. Huttunen, Jarkko Rautio

**Affiliations:** ^1^ School of Pharmacy University of Eastern Finland Kuopio Finland; ^2^ Medicinal Chemistry University of Florida College of Pharmacy Gainesville FL USA

**Keywords:** bioavailability, ketamine, oral administration, pharmacokinetics, prodrug

## Abstract

Ketamine, a rapid‐acting *N*‐methyl‐D‐aspartate (NMDA) receptor antagonist, has therapeutic potential beyond anesthesia, including treatment‐resistant depression. However, its low oral bioavailability due to extensive first‐pass metabolism and high abuse potential limit outpatient use. This study describes the design, synthesis, and in vivo evaluation of a ketamine prodrug conjugated to tyrosine methyl ester via a hydrolytically sensitive tertiary carbamate linker to improve oral absorption, achieve sustained release, and reduce abuse risk. The prodrug displayed moderate aqueous solubility and good chemical stability at physiological pH but was rapidly metabolized in enzyme‐containing media via demethylation of the tyrosine methyl ester to a demethylated prodrug, with no detectable ketamine release in vitro. In vivo pharmacokinetic studies in mice demonstrated that the prodrug underwent rapid metabolic conversion, resulting in detectable, though low, levels of released ketamine in plasma, liver, and brain. However, ketamine release was limited, and oral administration yielded very low bioavailability. These findings indicate that while tertiary carbamate‐based prodrugs can undergo in vivo activation, the current design does not sufficiently promote ketamine release or systemic exposure. Further structural optimization is required to improve oral bioavailability and achieve therapeutically meaningful delivery of ketamine.

## Graphical abstract

This study describes the development of an orally optimized ketamine prodrug designed to improve pharmacokinetic properties, extend therapeutic efficacy, and minimize adverse effects and abuse liability.

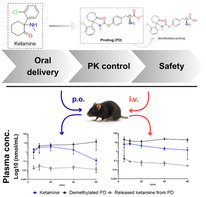

## 1 Introduction

Ketamine, a rapid‐acting general anesthetic and *N*‐methyl‐D‐aspartate (NMDA) receptor antagonist, has long been used for anesthesia induction and surgical procedures [1]. Originally developed in the 1960s as a safer alternative to phencyclidine, ketamine has gained increasing recognition beyond its anesthetic properties. More recently, its potential as a therapeutic agent for managing treatment‐resistant depression, bipolar disorder, and suicidal ideation has led to renewed interest in its pharmacokinetics and alternative routes of administration [2, 3, 4, 5, 6, 7].

**FIGURE 1:**
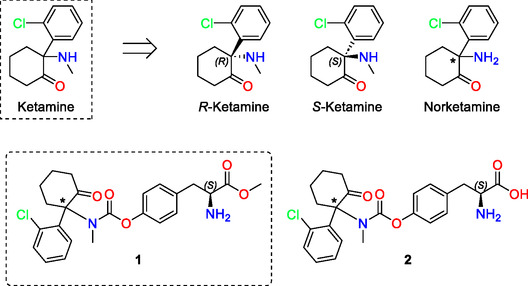
Structures of ketamine, *R‐*ketamine, *S*‐ketamine, norketamine, ketamine PD (**1**) and demethylated ketamine PD (**2**). The asterisk (*) denotes the racemic stereocenter in ketamine.

Chemically, ketamine is a racemic mixture composed of arketamine (*R*‐ketamine) and esketamine *(* *S‐*ketamine), both of which exhibit distinct pharmacological properties (Figure 1) [8, 9, 10]. As a 2‐(2‐chlorophenyl)‐2‐(methylamino)‐cyclohexane derivative with a chiral structure, ketamine exists as a pair of optical enantiomers due to an asymmetrical carbon at the C2 position. While racemic ketamine hydrochloride is commonly used in clinical settings, esketamine is the more active enantiomer in terms of NMDA receptor antagonism and is more potent than racemic ketamine [10, 11]. Enantiopure esketamine was introduced for medical use as an anesthetic in 1997 and as an antidepressant in 2019 [11, 12]. It is currently approved as an anesthetic in the European Union and as a nasal spray for treatment‐resistant depression in the United States and Canada. However, due to its hallucinogenic effects and misuse liability as a dissociative drug, esketamine is a controlled substance, necessitating careful monitoring in clinical use [11].

Traditionally, ketamine is administered intravenously (IV) or intramuscularly (IM) due to its high lipid solubility, rapid blood–brain barrier (BBB) penetration, and relatively short elimination half‐life [13, 14, 15, 16, 17]. However, these parenteral routes require clinical supervision, which limits their practicality in outpatient settings [18]. Oral administration presents a more convenient and scalable alternative but suffers from poor systemic availability, with bioavailability estimates as low as 17%–25% [17]. This limitation is largely attributed to extensive hepatic first‐pass metabolism, which rapidly converts ketamine into its primary metabolite, norketamine [19]. Interestingly, emerging evidence suggests that norketamine itself possesses pharmacological activity that may contribute to ketamine's efficacy in treating depression and pain. This raises the prospect of optimizing ketamine's oral formulation to enhance its therapeutic effects while mitigating undesirable side effects.

Another major concern surrounding ketamine therapy is its association with dissociative side effects and its high potential for abuse, particularly in recreational settings [20, 21]. The hallucinogenic and psychotomimetic effects of ketamine, while manageable in clinical settings, pose challenges for long‐term outpatient treatment. Challenges associated with ketamine have prompted the search for safer and more practical therapeutic alternatives. These limitations led us to hypothesize that by chemically modifying ketamine into an inactive precursor that undergoes controlled enzymatic activation in vivo, it may be possible to improve oral absorption, achieve steadier systemic levels, and reduce acute psychoactive effects as well as misuse potential.

This study aims to design, synthesize, and evaluate a ketamine prodrug (PD) optimized for oral administration (Figure 1). The primary objectives are to improve bioavailability, modulate pharmacokinetics to prolong therapeutic effects, and consequently reduce both acute adverse effects and the risk of misuse.

## 2 Materials and Methods

### 2.1 Materials

#### 2.1.1 General Synthetic Procedures

All reactions were performed with commercial reagents obtained from Sigma–Aldrich (St. Louis, MO, USA), Acros Organics (Waltham, MA, USA), Merck (Darmstadt, Germany), Thermo Fisher Scientific (Heysham, China), AmBeed (Arlington Hts, IL, USA), and AK Scientific (Union City, CA, USA). All solvents used in the reactions were anhydrous. Dichloromethane (DCM), *N*,*N‐*dimethylformamide (DMF), and tetrahydrofuran (THF) were dried over molecular sieves (4 Å) and were stored under an inert atmosphere. All reactions were performed under an inert atmosphere of argon or nitrogen unless otherwise specified. Reactions were monitored by thin‐layer chromatography using aluminum sheets coated with silica gel 60 F245 (0.24 mm) with a suitable visualization agent. Purifications by flash chromatography (BUCHI, Sepacore flash systems X10) were performed on silica gel 60 (0.063–0.200 mm mesh) cartridges. The ^1^H and ^13^C‐NMR spectra were recorded on a Bruker Avance HD III 600 spectrometer, equipped with 5 mm cryogenically cooled BBO probehead and operating at 600.18 and 150.93 MHz, respectively. All NMR experiments were measured at 298 K. Chemical shifts are reported in ppm on the *δ* scale from an internal standard of solvent MeOD‐*d*_4_ referenced to 3.31 (^1^H) and 49.00 (^13^C) ppm). The spectra were processed from the recorded FID files with either TOPSPIN 2.1 or Mestrenova software. Following abbreviations are used: s, singlet; d, doublet; dd, doublet of doublets; ddd, doublet of doublets of doublets; t, triplet; m, multiplet. Coupling constants are reported in Hz. The high‐resolution ESI‐MS spectra were recorded by an Agilent 1290 Infinity LC system coupled with an Agilent G6540A quadrupole time‐of‐flight mass spectrometer with an electrospray ionization (ESI) source (Agilent Technologies, Palo Alto, CA, USA).

#### 2.1.2 Synthesis of the PD

##### 2.1.2.1 Methyl (*2S*)‐2‐((*tert*‐butoxycarbonyl)amino)‐3‐(4‐(((1‐(2‐chlorophenyl)‐2‐oxocyclohexyl)(methyl) carbamoyl)oxy)phenyl)propanoate (3)

Under an inert atmosphere, triphosgene (200 mg, 0.68 mmol, 1.2 eq.) was suspended into a round bottom flask and DCM (5 mL) was added at room temperature. To this mixture, a solution of methyl (*tert*‐butoxycarbonyl)‐L‐tyrosinate (334 mg, 1.13 mmol, 2.0 eq.) in DCM (5 mL) was added at 0°C. To the crude reaction mixture, a solution of diisopropylethylamine (0.59 mL, 3.39 mmol, 6.0 eq.) in THF (10 mL) was slowly added at 0°C, over 30 min and stirred on ice‐bath to 0°C for 45 min. To the reaction mixture, racemic‐ketamine HCl (186 mg, 0.68 mmol, 1.0 eq.) dissolved in DCM (5 mL) and DIPEA (0.12 mL, 0.68 mmol, 1.2 eq.) was added at 0°C. The red reaction mixture was stirred at 0°C and allowed to warm to room temperature and stirred at room temperature for 48 h. Upon completion, the reaction mixture was concentrated, adsorbed onto silica, and purified by combi‐flash eluting with DCM:MeOH (100:0–0:100) over 45 min. The desired product **3** was obtained as a slight brown solid (543 mg, 0.97 mmol, 86%). TLC (*R*_f_) = 0.90 (DCM/MeOH, 9:1). ^1^H NMR (600 MHz, MeOD) *δ* 7.67 (dd, *J* = 7.9, 1.5 Hz, 1H), 7.39 (tdd, *J* = 7.8, 6.7, 1.2 Hz, 2H), 7.34–7.27 (m, 3H), 7.21 (d, *J* = 8.2 Hz, 2H), 4.38 (dd, *J* = 9.1, 5.5 Hz, 1H), 3.69 (s, 3H), 3.13 (dd, *J* = 13.9, 5.6 Hz, 1H), 3.00–2.90 (m, 1H), 2.84–2.79 (m, 1H), 2.51–2.46 (m, 1H), 2.45–2.38 (m, 1H), 2.04 (s, 3H), 2.02–2.00 (m, 1H), 1.89–1.82 (m, 2H), 1.77–1.69 (m, 2H), 1.39 (s, 9H), 1.30–1.28 (m, 1H). ^13^C NMR (151 MHz, MeOD) *δ* 210.71, 174.02, 157.81, 153.70, 151.43, 138.68, 136.80, 134.82, 132.20, 131.49, 131.11, 130.23, 128.10, 122.00, 80.67, 71.37, 56.42, 52.65, 40.57, 39.70, 37.99, 29.33, 28.98, 28.65, 22.69.

##### 2.1.2.2 (2S)‐3‐(4‐(((1‐(2‐chlorophenyl)‐2‐oxocyclohexyl)(methyl)carbamoyl)oxy)phenyl)‐1‐oxopropan‐2‐aminium (1) trifluoroacetate salt

The ketamine‐conjugate **3** (225 mg, 0.4 mmol, 1.0 eq.) was dissolved in DCM (5 mL) and cooled to 0°C. Excess TFA (0.16 mL, 2.01 mmol, 5.0 eq.) was added to the reaction mixture and stirring was continued at room temperature over 24 h. Additional amount of TFA (0.16 mL, 2.01 mmol, 5.0 eq.) was added to the reaction mixture, which was stirred at room temperature for another 24 h. Upon completion, the reaction mixture was concentrated, adsorbed onto silica, and purified by combi‐flash eluting with DCM:MeOH (100:0–0:100) over 45 min. The desired PD (**1**) was obtained as a slight brown oil (122 mg, 0.21 mmol, 53%). TLC (*R*_f_) = 0.53 (DCM/MeOH, 9:1). ^1^H NMR (600 MHz, MeOD) *δ* 7.49 (dd, *J* = 7.8, 2.2 Hz, 1H), 7.38–7.31 (m, 2H), 7.31–7.25 (m, 2H), 7.22 (d, *J* = 7.7 Hz, 1H), 7.18–7.04 (m, 2H), 4.34–4.28 (m, 1H), 3.83 (s, 3H), 3.30–3.26 (m, 1H), 3.22 (s, 3H), 3.17–3.09 (m, 1H), 2.87–2.76 (m, 1H), 2.61–2.45 (m, 2H), 2.12–2.03 (m, 1H), 1.95–1.85 (m, 2H), 1.82–1.74 (m, 1H), 1.41–1.36 (m, 1H), 1.28–1.19 (m, 1H). ^13^C NMR (151 MHz, MeOD) *δ* 170.44, 157.65, 133.83, 133.13, 131.78, 131.58, 131.53, 130.38, 128.00, 123.80, 123.71, 122.90, 76.98, 55.21, 53.69, 41.69, 36.71, 33.07, 28.35, 22.99. MS (ESI—positive mode): For C_24_H_28_ClN_2_O_5_ [M + H] calculated: 459.1681; found: 459.1676.

#### 2.1.3 Solubility

The aqueous solubility of ketamine (hydrochloride) and the PD was determined at room temperature. Each compound (1 mg) was added to 1 mL of 50 mM sodium phosphate buffer (pH 6.5) and shaken overnight. The samples were centrifuged, and 50 µL of the supernatant was carefully collected for UV–high‐performance liquid chromatography (HPLC) analysis, as described below. The concentration of the dissolved compound was quantified using an external standard method.

#### 2.1.4 Chemical and Metabolic Stability

The chemical stability of ketamine and its PD was studied in sodium phosphate buffer (pH 6.8) at 37°C. Sampling (50 µL) was performed at several time points, and each sample was mixed with 50 µL of acetonitrile (1:1, v/v).

The metabolic stability of ketamine and its PD was evaluated in human and rat liver microsomes and serum at 37°C. The liver microsome incubation mixtures consisted of liver microsomes (50 µL, 1 mg/mL final protein concentration), NADPH (200 µL), 20 mM potassium phosphate buffer (pH 7.4, 748.5 µL), and a stock solution of ketamine or PD (1.5 µL of a 10 mg/mL stock solution in DMSO, final concentration 54.7 µM for ketamine, 32.7 µM for PD). The solutions were incubated in Eppendorf vials on a Thermomixer 5436 (Eppendorf, Hamburg, Germany) at 11 × 100 rpm at 37°C. For the serum experiments, the same stock solution (10 µL) was added to serum (800 µL), diluted with 200 µL of 50 mM sodium phosphate buffer, resulting in final concentrations of 361 µM for ketamine and 216 µM for the PD.

Sampling (50 µL) was performed at several time points. Proteins in the samples were precipitated with ice‐cold acetonitrile (100 µL), and the samples were centrifuged at 12,000 × *g* for 5 min at +4°C. The supernatants were collected and analyzed using the UV–HPLC method described below. Each experiment was performed three times, with *n* = 3.

#### 2.1.5 HPLC Analysis

Samples from solubility, chemical, and metabolic stability studies were analyzed using an Agilent 1100 Series HPLC system (Agilent Technologies, Inc., Santa Clara, CA, USA) equipped with a binary pump, autosampler, and photodiode array (PDA) detector. A ZORBAX RR Eclipse Plus C18 column (3.0 × 100 mm, 3.5 µm; Agilent Technologies, Palo Alto, CA, USA) was used for the analysis.

Analytes were eluted using an isocratic method with a flow rate of 1.0 mL/min. The mobile phase consisted of 0.1% formic acid in water (A) and acetonitrile (B) in a ratio of 85:15 (v/v). The column temperature was maintained at 40°C, and detection was carried out at a wavelength of 210 nm.

The HPLC method was validated to be accurate (100 ± 10%), precise (RSD < 15%), and specific within the concentration range of 2.5–50 µM. The lower limit of detection was 2.5 µM.

#### 2.1.6 Pharmacokinetic Study

The pharmacokinetic profiles of ketamine and its PD were evaluated in mice following both intravenous and oral administration. Plasma and brain concentrations were measured and compared to assess oral bioavailability and brain penetration. The aim of the study was to evaluate the potential of a ketamine PD optimized for oral delivery.

All animal experiments were conducted under license ESAVI‐2020‐025070, approved by the Finnish Project Authorization Board, and in accordance with the European Community Guidelines (Directive 2010/63/EU) and the Guide for the Care and Use of Laboratory Animals. Studies were carried out during the light phase, and every effort was made to minimize both the number of animals used and their suffering.

Eight‐ to 9‐week‐old healthy male mice (C57BL/6JOlaHsd; 30 ± 5 g), obtained from Envigo (The Netherlands), were used in the experiments. The animals were housed in well‐ventilated stainless‐steel cages under a 12‐h light/dark cycle, with free access to tap water and food pellets.

Ketamine was dissolved in phosphate buffer (pH 7.4), while the PD was dissolved in 5% DMSO and 30% Solutol in phosphate buffer (pH 7.4). The compounds were administered orally (PO) (10 mL/kg) or intravenously (IV) (5 mL/kg) at a dose corresponding to 10 mg/kg of ketamine equivalent. At selected time points (5, 10, 20, 40, and 60 min postdose), mice were anesthetized with intraperitoneal pentobarbital (120 mg/kg, Euthoxin Vet, Chanelle Pharmaceuticals Manufacturing Ltd., Ireland), followed by a short transcardial perfusion with ice‐cold physiological saline for 1 min to remove excess blood from the tissues. Whole blood samples were collected via cardiac puncture prior to perfusion. Liver and brain samples were collected at each time point. To obtain plasma, whole blood samples were centrifuged in heparinized tubes at 2000 × *g* for 10 min at 4°C. All samples were stored at −80°C until analysis.

#### 2.1.7 Sample Analysis of PK Samples

To quantify drug concentrations in the tissue samples, the snap‐frozen tissues were weighed into 2 mL Bead Ruptor bead beating tubes (Omni International, Kennesaw, GA, USA) prefilled with 1.4 mm ceramic beads. Milli‐Q water was added to the tubes 1:3 (w/v), and samples were homogenized at 4°C using a bead mill homogenizer (Omni Bead Ruptor 24 Elite homogenizer with a BR Cryo cooling unit, Omni International, Kennesaw, GA, USA).

The proteins in the tissue homogenates and plasma samples were precipitated by diluting the samples 1:3 (v/v) with acetonitrile containing 200 nM labetalol and 0.1% formic acid. Samples were incubated at 4°C overnight, followed by centrifugation at 16,000 × *g* for 10 min at 4°C. The supernatants were diluted 1:1 (v/v) with milli‐Q water and transferred into HPLC vials for quantification by the liquid chromatography coupled with mass spectrometry (LC–MS) method described below. The drug standards for each compound were prepared separately in the same manner.

From the LC–MS results, time‐concentration profiles of ketamine and PD were calculated for the studied tissues using GraphPad Prism software (version 10.3, San Diego, CA, USA). The area under the curve (AUC) was calculated using the linear trapezoidal method, providing simultaneously the numeric pharmacokinetic parameters for peak concentration (*C*_max_) and peak timepoint (*t*_max_).

#### 2.1.8 Quantification of the Drug Concentrations by LC–MS

The amounts of ketamine, its PD, and demethylated PD were determined using the LC–MS method. The compounds were separated using the HPLC system (1200 Series Rapid Resolution LC System, Agilent Technologies, Santa Clara, CA, USA) equipped with a reversed‐phase column (Zobrax XDB‐C18 RRHT, 50 mm × 4.6 mm, 1.8 μm; Agilent Technologies, Santa Clara, CA, USA) and the mobile phase of water (A) and acetonitrile (B), both containing 0.1% formic acid. The studied compounds were separated using the following mobile phase gradient: 0–1.5 min: 20% → 75% B, 1.5–5 min: 75% B, 5–5.5 min: 75% → 20% B, 5.5–8 min: 20% B. The mobile phase flow rate was 0.4 mL/min, the column temperature was 40°C, and the sample injection volume was 5 µL.

A triple quadrupole mass spectrometry (QQQ 6410, Agilent Technologies, Santa Clara, CA, USA) with an ESI source was used for detection. The multiple reaction monitoring (MRM) data acquisition was performed in positive electrospray ionization (ESI+) mode with the following conditions: drying gas (nitrogen) flow rate of 8 L/min with a temperature of 300°C, nebulizer pressure of 40 psi, and the capillary voltage of 4 kV. The fragmentor voltage was 70 V for labetalol (internal standard), 100 V for ketamine, and 120 V for PD with and without methylation. Following MRM transitions were recorded with the collision energies in brackets: 329.0 → 294.0 (10 V) and 162.0 (10 V) for labetalol, 238.1 → 179.0 (12 V) and 125.0 (25 V) for ketamine, 459.2 → 253.2 (7 V) and 179.1 (20 V) for PD, and 445.1 → 239.1 (5 V) and 207.0 (14 V) for demethylated PD. The data acquisition software was the Agilent MassHunter Workstation software (version B.03.00), and the Quantitative Analysis (B.09.00) software was used for data processing and analysis. The LC–MS method was validated to be highly selective, linear (*R* ^2^ > 0.995), accurate (100 ± 15%), and precise (RSD < 15%) over the used calibration range of 2–5000 nM. The lower limit of quantification (LLOQ) for all studied compounds was 2 nM.

## 3 Results and Discussion

### 3.1 Design of the PD

Amino acid PDs have an established commercial and regulatory track record, which facilitates their development and clinical translation [22]. Among them, amino acid amides and carbamates present a great opportunity for designing sustained‐release PDs, as these bonds are generally more resistant to chemical and enzymatic hydrolysis than the corresponding ester bonds [23, 24]. In this study, a ketamine tyrosine PD was engineered by conjugating ketamine to tyrosine methyl ester via a hydrolytically sensitive tertiary carbamate linker. This design utilizes the phenolic hydroxyl group at the *para*‐position of tyrosine as a natural conjugation site. Additionally, methylation of the tyrosine carboxylic acid prevents zwitterion formation, which could otherwise impair membrane permeability following oral administration [25]. As a result, the esterified form is expected to exhibit improved oral absorption compared to the free carboxylic acid, enhancing its potential as an orally bioavailable PD.

We hypothesized that using a carbamate linkage would provide a promising strategy for achieving sustained release of ketamine by maintaining therapeutic plasma concentrations while minimizing the risk of abuse and side effects typically associated with rapid onset and high peak exposures. Tertiary carbamate groups are known to undergo base‐catalyzed hydrolysis via a BAC2 (bimolecular base‐catalyzed addition–elimination) mechanism, facilitated through nucleophilic or general base catalysis [26]. Although tertiary carbamates are generally considered more hydrolytically stable than their secondary counterparts due to increased steric hindrance and the absence of an N—H proton, they are also susceptible to enzymatic hydrolysis. In our previous study, the dopamine–tyrosine PD (Dopa‐CBT), which incorporated a hydrolyzable secondary carbamate, demonstrated slow release of dopamine via both chemical and enzymatic hydrolysis in pH 7.4 buffer, rat plasma, and 10% rat liver homogenate [27].

In the present case, despite the use of a more stable tertiary carbamate, we hypothesized that enzymatic activity, particularly from carboxylesterases and other tissue‐expressed hydrolases, could still promote PD activation. The selection of a tertiary carbamate linker was therefore a deliberate attempt to strike a balance between chemical stability and susceptibility to enzymatic activation under physiological conditions, enabling a controlled and extended‐release profile of ketamine.

**SCHEME 1:**
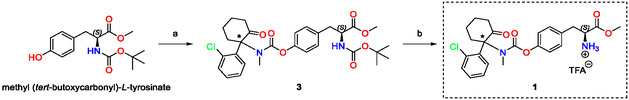
Synthesis of PD. Reagents and conditions: (a) (i) triphosgene, DIPEA, THF, DCM, 0°C, 45 min and (ii) racemic‐ketamine HCl, DIPEA, DCM, 0°C to room temperature, 48 h (86%); (b) TFA, DCM, 48 h, purification by combi‐flash chromatography (53%). The asterisk (*) denotes the racemic stereocenter in ketamine.

The synthesis of *para*‐conjugated tyrosine PD (**1**) was completed in two linear steps (Scheme 1). With a properly protected amino acid section, the commercially available methyl (*tert*‐butoxycarbonyl)‐L‐tyrosinate served as the ideal starting material for synthesis. Treatment of the protected amino acid with triphosgene in the presence of DIPEA in THF/DCM gave the intermediate phenylchloroformate. This in situ formed chloroformate was further reacted in the same reaction flask with the ketamine hydrochloride to afford the key intermediate **3** in good yield (86%). Finally, the selective removal of the Boc group was achieved by the treatment of intermediate **3** with excess of TFA over 2 days, resulting in the formation of the desired PD (**1**, 53%).

### 3.2 Physicochemical Properties and In Vitro Stability

The aqueous solubility of the PD was determined to be 0.5 mg/mL in phosphate buffer at pH 6.5 (Table 1). In comparison, ketamine showed a solubility greater than 1 mg/mL under the same conditions. The moderate solubility of the PD aligns with its increased lipophilicity (calculated log*P* values: 2.9 for ketamine and 3.5 for the PD, ChemDraw 22.2.0.3300), resulting from the methylation of the tyrosine carboxyl group and the incorporation of a bulky, tertiary carbamate moiety [25].

**TABLE 1:** Aqueous solubility (mg/mL), chemical stability (*t*_1/2_, h), and in vitro plasma (human *t*_1/2_, h; rat *t*_1/2_, h) and liver microsomal (*t*_1/2_, min) half‐lives of ketamine and its PD (mean ± SD, *n *= 3).

	Aqueous solubility pH 6.5, mg/mL	Chemical stability pH 7.4 *t*_1/2_, h	Human plasma *t*_1/2_, h	Rat plasma *t*_1/2_, min	Human liver microsomes *t*_1/2_, min	Rat liver microsomes *t*_1/2_, min
Ketamine	>1	stable for 8 h	stable for 6 h	stable	Some degradation occurs in 6 h	70.0 ± 14.5
PD	0.53 ± 0.05	7.6 ± 0.7	2.6 ± 0.1	0.8 ± 0.2	3.4 ± 0.3	0.50 ± 0.11

Chemical stability studies showed that the PD degraded with a half‐life of 7.6 h (*n* = 3) in an aqueous solution at pH 7.4°C and 37°C (Table 1). However, during the 8‐h incubation, no ketamine was detected by UV–HPLC. A single degradation product, which eluted slightly earlier than the intact PD, was consistently observed and is presumed to be a demethylated PD (Figure 1). Ketamine, when present, eluted much earlier in the chromatogram but was not detected under these conditions. These results indicate that the PD remains reasonably stable in aqueous buffer, supporting the idea that the tertiary carbamate linker protects from spontaneous hydrolysis in the absence of enzymatic activity [23].

However, the PD was rapidly metabolized in all enzyme‐containing media tested, using both rat and human matrices, while accounting for the well‐known interspecies differences in PD‐metabolizing enzymes (Table 1) [28, 29]. In liver microsome incubations, the half‐life was approximately 0.5 min in rat and 3.4 min in human microsomes (*n* = 3). Ketamine itself was not detected by UV–HPLC. Instead, a metabolite with a slightly shorter retention time was consistently observed. Rapid mass spectrometry analysis of this product revealed an *m*/*z* value of 445.0, consistent with the proposed demethylated PD. These findings suggest that enzymatic activity readily initiates biotransformation of the PD, yet the anticipated ketamine release was not observed under the tested in vitro conditions.

This metabolic behavior may reflect a limitation in the enzymatic cleavage pathway required to fully release ketamine. Tertiary carbamates are generally more stable due to steric hindrance and the absence of an N—H proton, which would otherwise allow E1cB‐mediated hydrolysis, but they remain susceptible to enzymatic hydrolysis, particularly by carboxylesterases and related hydrolases. Our design aims to balance sufficient chemical stability for oral administration with enzymatic activation in vivo. The absence of detectable ketamine release in vitro, despite rapid metabolism of the PD, suggests that the initial enzymatic ester cleavage generates a stable intermediate (e.g., the demethylated PD), which may require further transformation under more prolonged or specific conditions.

### 3.3 In Vivo Pharmacokinetics

Despite the PD's moderate aqueous solubility and limited ketamine release in vitro, in vivo studies were conducted to assess its pharmacokinetics and metabolic fate under physiologically relevant conditions. While in vitro models are valuable for initial screening, including liver microsomes that contain metabolizing enzymes such as esterases bound to the endoplasmic reticulum of hepatocytes, they do not replicate the enzymatic activity of complex biological environments. Therefore, in vivo evaluation was necessary to determine whether the PD could release ketamine in a therapeutically relevant manner.

Ketamine is traditionally administered intravenously or intramuscularly due to its high lipid solubility, rapid BBB penetration, and short elimination half‐life [30, 31]. However, these parenteral routes require clinical supervision, making them less suitable for outpatient use. Oral administration offers a more practical alternative, but suffers from poor systemic bioavailability, reported as low as 17%–25%, mainly due to extensive hepatic first‐pass metabolism that rapidly converts ketamine into its primary metabolite, norketamine. In the present study, norketamine concentrations were not monitored, and thus its potential contribution to the observed pharmacokinetic profile could not be assessed.

The time points (5, 10, 20, 40, and 60 min) were selected based on the rapid clearance of ketamine and were applied to both ketamine and the PD. In mice, the elimination half‐life of ketamine typically ranges from 15 to 25 min, depending on the route of administration, dose, and experimental conditions [32, 33, 34]. The first sampling was conducted at 5 min postdose. Given ketamine's rapid distribution, particularly after intravenous administration, peak concentrations are typically reached within 1–2 min, so that for the oral group, the 5 min time point likely represents the early absorption phase.

**FIGURE 2:**
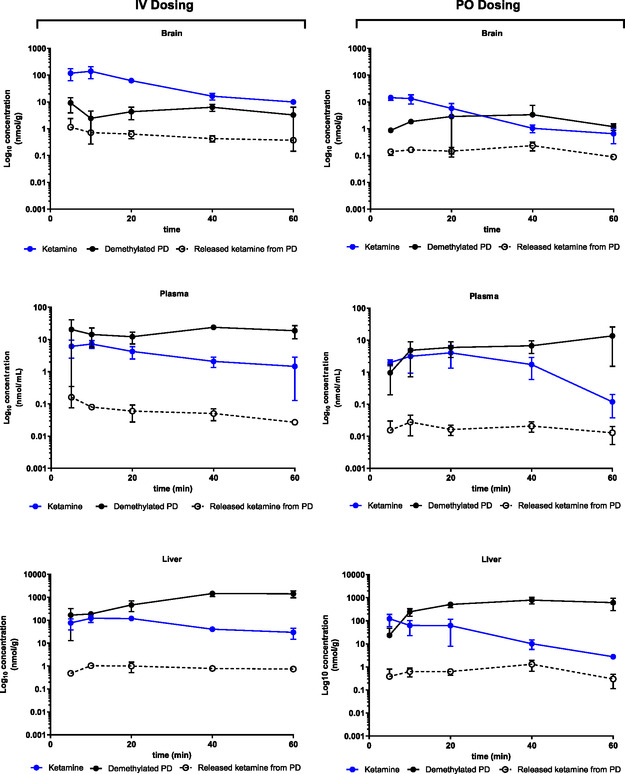
In vivo pharmacokinetic profiles of ketamine (circle with solid blue line), demethylated PD (circle with solid black line), and released ketamine from PD (circle with black dashed line) in mouse brain, plasma, and liver tissue following IV and PO dosing. Results are expressed as mean ± SD (*n* = 3).

Following intravenous administration, peak ketamine concentrations were observed at 10 min postdose in all studied tissues: plasma (7.23 nmol/mL), brain (140 nmol/g), and liver (121 nmol/g) (Figure 2, Table 2). After oral administration, peak plasma concentration reached 20 min (4.06 nmol/mL), while both brain and liver concentrations peaked at 5 min: 14.6 nmol/g (brain) and 122 nmol/g (liver). Although ketamine is highly lipophilic and rapidly crosses the BBB due to the high perfusion of the brain, its lower concentration in brain tissue compared to the liver can be explained by rapid redistribution into peripheral tissues and high hepatic uptake. Additionally, the markedly lower brain concentration following oral dosing compared to intravenous administration is likely due to reduced systemic availability caused by first‐pass metabolism in the liver and slower, less efficient absorption from the gastrointestinal tract, resulting in lower plasma levels and consequently less drug available to penetrate the brain.

**TABLE 2:** In vivo pharmacokinetic parameters of ketamine, demethylated PD, and released ketamine from PD following IV and PO dosing.

	Ketamine		Released ketamine from PD		Demethylated PD	
PK parameter	IV	PO	IV	PO	IV	PO
AUC_plasma_, min × nmol/mL	189	125	3.18	1.04	1005	399
AUC_brain_, min × nmol/g tissue	2707	251	30	9.35	265	138
AUC_liver_, min × nmol/g tissue	3974	1932	47.2	43.9	51 919	31 217
AUC_brain/plasma_	14.3	2	9.44	8.98	0.26	0.35
AUC_liver/plasma_	21	15.4	14.8	42.2	51.7	78.3
*C*_max_ plasma, nmol/mL	7.23	4.06	0.161	0.028	23.9	13.7
*t*_max_ plasma, min	10	20	5	10	40	60
*C*_max_ brain, nmol/g tissue	140	14.6	1.14	0.235	9.92	3,35
*t*_max_ brain, min	10	5	5	40	5	40
*C*_max_ liver, nmol/g tissue	121	122	1.04	1.29	1451	780
*t*_max_ liver, min	10	5	10	40	40	40

The intact PD was not detectable in any of the samples. However, its demethylated PD and the released ketamine were detected, although the concentration of the released ketamine was lower compared to ketamine itself. Following intravenous (IV) administration, the demethylated PD reached peak concentrations of 23.9 nmol/mL in plasma and 1451 nmol/g in the liver at 40 min, and 9.92 nmol/g in the brain at 5 min. After oral (PO) administration, the peak concentrations were 13.7 nmol/mL in plasma at 60 min, and in both brain and liver at 40 min: 3.35 nmol/g (brain) and 780 nmol/g (liver).

The ketamine released from the PD also showed measurable concentrations. After IV dosing, peak levels were reached at 5 min in both plasma (0.16 nmol/mL) and brain (1.14 nmol/g), while in the liver, the peak (1.04 nmol/g) occurred at 10 min. In contrast, after PO administration, peak concentrations were 0.028 nmol/mL in plasma at 10 min, and at 40 min in both brain (0.235 nmol/g) and liver (1.29 nmol/g). The bioavailability of ketamine was approximately 66%, while ketamine released from the PD was around 32% (AUC_released_, PO/AUC_released_, IV). These results should be interpreted cautiously due to the relatively short experimental timeframe. This approach focuses directly on the pharmacokinetic behavior of the ketamine fraction released from the PD, rather than the parent ketamine administered separately, allowing us to isolate and evaluate the rate and extent of ketamine liberation as well as the sustained‐release properties that were the key focus of this study. In contrast, ketamine‐relative bioavailability (AUC_released_, PO/AUC_ketamine_, _IV)_ was below 1%, reflecting the inherent limitations of oral ketamine, such as extensive first‐pass metabolism, and does not fully address the main pharmacological question of this study. Although not directly addressed in the present study, it is worth noting that related amino acid PDs bearing both free amino and carboxyl groups, such as Dopa‐CBT, have been shown to act as LAT1 substrates. Given that LAT1 is expressed at the BBB [35, 36], it may have contributed to the distribution profile, especially the brain, observed for the demethylated PD.

These in vivo results demonstrate that the intact PD was rapidly metabolized into a demethylated PD and subsequently to ketamine, with detectable levels in both plasma and tissues. The metabolic conversion occurred more rapidly and to a greater extent following IV administration compared to oral dosing.

Overall, ketamine achieved higher concentrations in all studied tissues following direct administration compared with levels observed after dosing with its PD, with significantly greater exposure observed after intravenous administration. For instance, the brain‐to‐plasma area under the curve (AUC) ratios were approximately 7 times higher following IV dosing compared to oral administration of ketamine itself. The low oral bioavailability of ketamine reported in the literature is primarily due to extensive first‐pass hepatic metabolism [31]. Additionally, ketamine is a known substrate of the efflux transporters P‐glycoprotein (Pgp) and breast cancer resistance protein (Bcrp), which may affect its systemic distribution and contribute to variability in therapeutic responses [34].

Notably, when examining the AUC brain/plasma ratios for the ketamine released from the PD, the values were comparable between IV and PO administration. Although the absolute concentrations were lower, the release appeared stable across both routes, suggesting a sustained release profile and the potential for a prolonged pharmacological effect.

Building on these results, ketamine was designed as a PD conjugated to tyrosine methyl ester via a hydrolytically sensitive tertiary carbamate linker, balancing chemical stability with enzymatic lability for in vivo activation under physiological conditions. The design strategy relied on the phenolic hydroxyl group of tyrosine for conjugation and the methylation of its carboxylic acid to improve membrane permeability by preventing zwitterion formation [25]. This approach enabled controlled release and sustained therapeutic action while mitigating risks associated with rapid systemic exposure.

The results indicate that the PD exhibits moderate aqueous solubility, consistent with its increased lipophilicity resulting from structural modifications. Its chemical stability under physiological conditions was sufficient to prevent premature degradation, supporting oral administration. These properties align with key requirements for oral PDs, balancing formulation stability with adequate membrane permeability to support oral administration.

However, in vitro studies revealed that although the PD was rapidly metabolized in enzyme‐containing media, particularly in rat and human liver microsomes, no ketamine was released under these conditions. Instead, a consistent demethylated PD was observed, suggesting that the metabolic activation proceeds through a multistep pathway.

In vivo pharmacokinetic studies confirmed that the PD was absorbed and metabolized in mice, with both the demethylated PD and released ketamine detected in plasma, liver, and brain. Notably, while absolute concentrations of released ketamine were lower after oral administration compared to intravenous dosing, the presence of ketamine in target tissues, particularly the brain, supports successful metabolic activation in vivo. This highlights that in vitro models may not fully capture the metabolic activation occurring in vivo. Moreover, the comparable brain‐to‐plasma AUC ratios for ketamine released from the PD across IV and oral routes indicate a stable and extended release profile, supporting the initial design hypothesis.

Nevertheless, the relatively low bioavailability of released ketamine following oral dosing reflects a limitation of this approach. The results suggest that while enzymatic cleavage occurs in vivo, the process may be incomplete or rate‐limiting, potentially due to the steric hindrance inherent in tertiary carbamate structures. This highlights a need for further optimization to improve the efficiency of ketamine liberation from the demethylated PD.

## 4 Conclusions

In conclusion, while the PD fulfills several key criteria for oral administration, including aqueous stability, enhanced membrane permeability, and in vivo metabolic activation, it falls short in achieving high systemic exposure to ketamine. These findings underscore the utility of tertiary carbamate‐based designs in achieving controlled release but also point to the complexity of predicting enzymatic bioconversion pathways. Future iterations should focus on enhancing enzymatic cleavage efficiency to improve bioavailability, while preserving the favorable pharmacokinetic characteristics demonstrated in this study. Although this PD design provides a potential starting point for developing safer and more practical oral ketamine therapies, further structural optimization to improve release efficiency and systemic exposure should be prioritized before proceeding to longer and more comprehensive pharmacokinetic studies involving extended sampling and detailed metabolite analysis.

## Author Contributions

The manuscript was written through the contributions of all authors. **Kenneth B. Sloan**, **Kristiina M. Huttunen**, and **Jarkko Rautio** participated in the research design. **Juulia Järvinen**, **Santosh Kumar Adla**, **Janne Tampio**, and **Aaro Jalkanen** conducted experiments. **Juulia Järvinen**, **Santosh Kumar Adla**, **Janne Tampio**, **Aaro Jalkanen**, and **Jarkko Rautio** contributed to data analysis and manuscript writing. **Aaro Jalkanen**, **Kenneth B. Sloan**, **Kristiina M. Huttunen**, and **Jarkko Rautio** revised the manuscript. All authors have read and approved the final version of the manuscript.

## Conflicts of Interest

The authors declare no conflicts of interest.

## Supporting information

Supplementary Material

## Data Availability

Data will be made available on request.
