# Supplementary material for: A Novel Tertiary Carbamate Prodrug Strategy to Overcome Metabolic Barriers in Oral Ketamine Delivery
Source: ChemMedChem. 2026 Feb 3;21(2):e202500856. doi: 10.1002/cmdc.202500856 (PMC12867590; doi:10.1002/cmdc.202500856)

^{1}H-NMR (600 MHz, MeOD) of the intermediate **3**

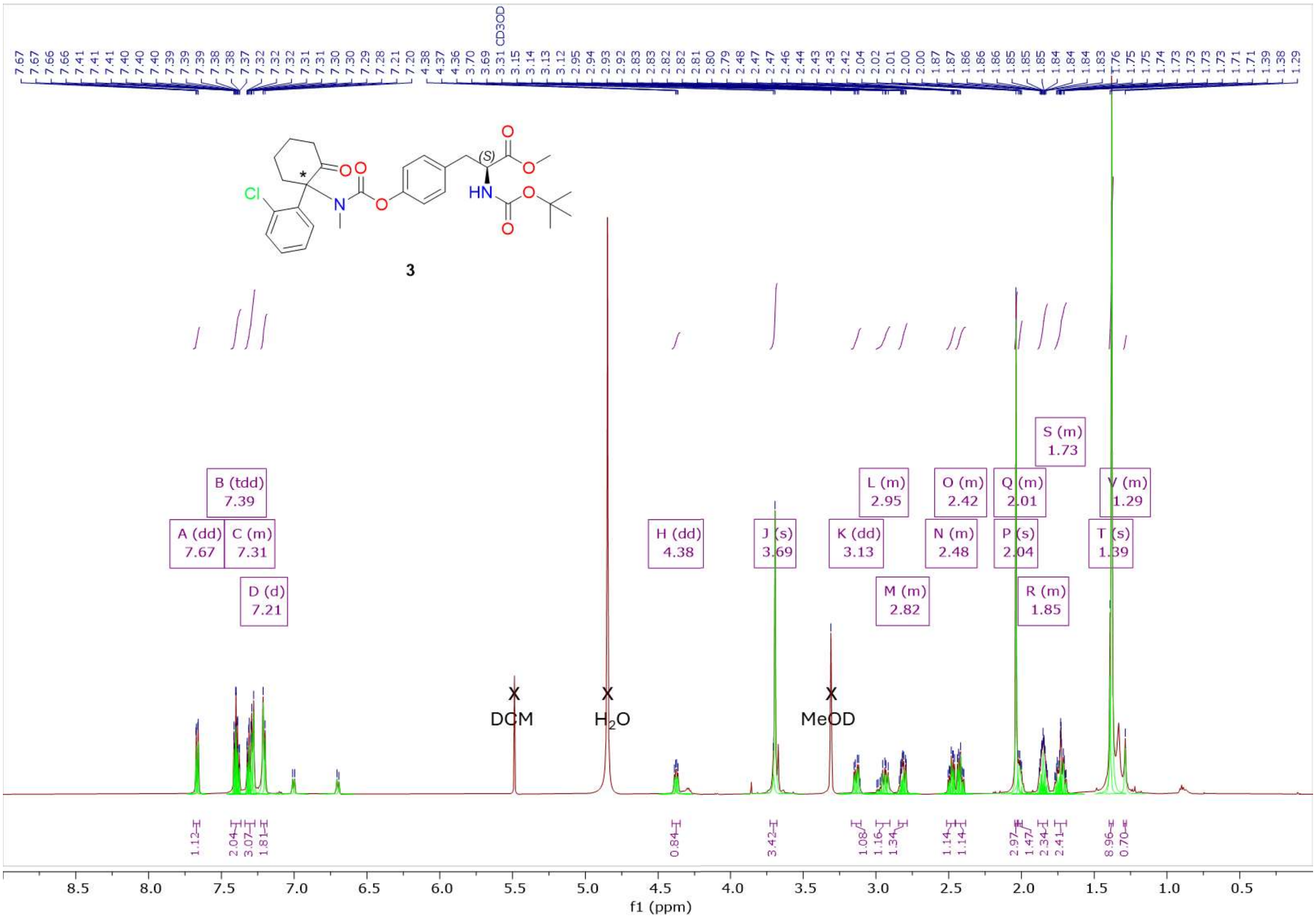

^{13}C-NMR (151 MHz, MeOD) of the intermediate **3**

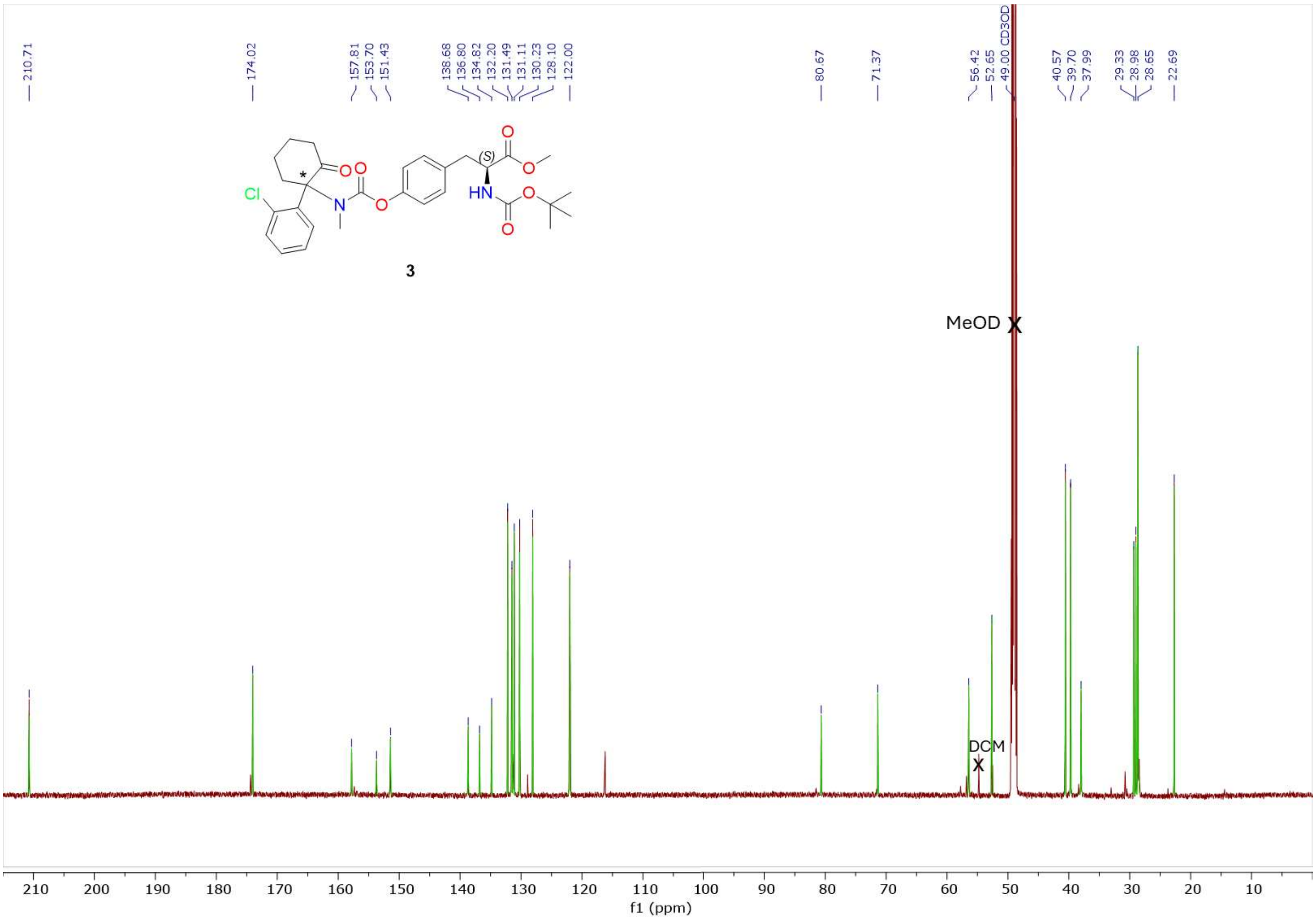

^{1}H-NMR (600 MHz, MeOD) of the Prodrug **1**

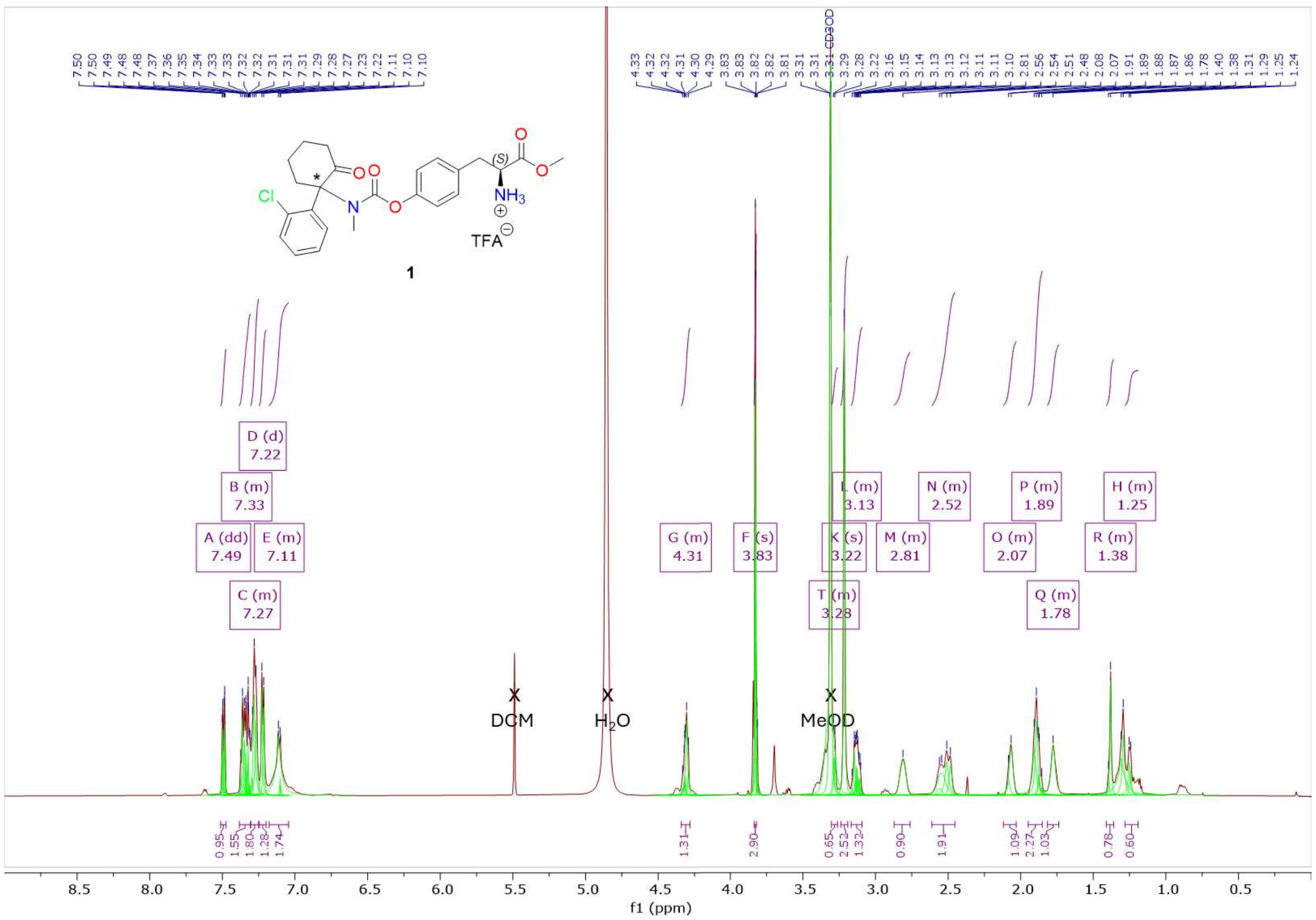

^{13}C-NMR (151 MHz, MeOD) of the Prodrug **1**

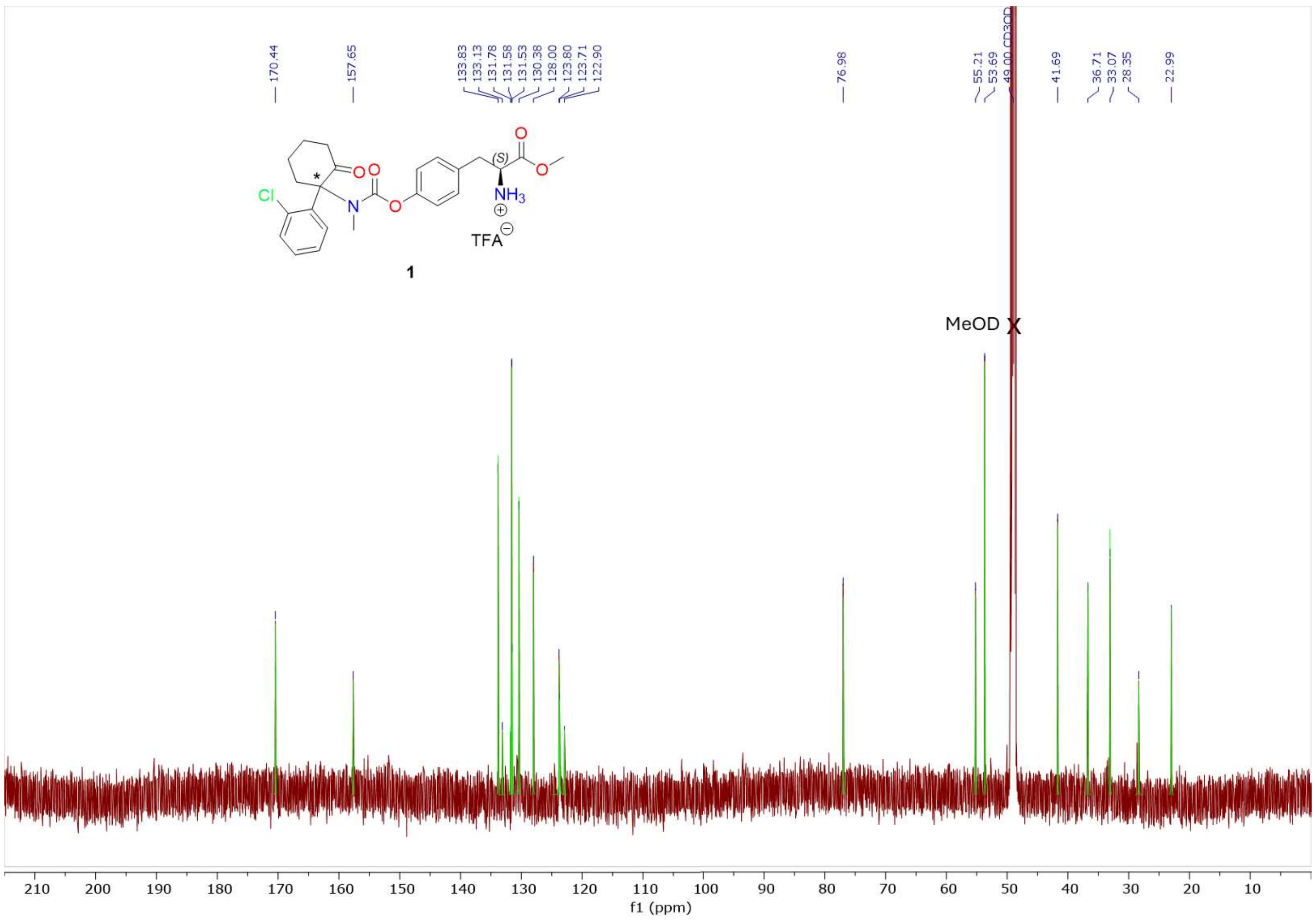

HR-MS of the Prodrug **1**

The sample was analyzed by liquid chromatography coupled with electrospray ionization mass spectrometry. Calculated $[M+H]^+$: 459.1681, found $[M+H]^+$: 459.1676.

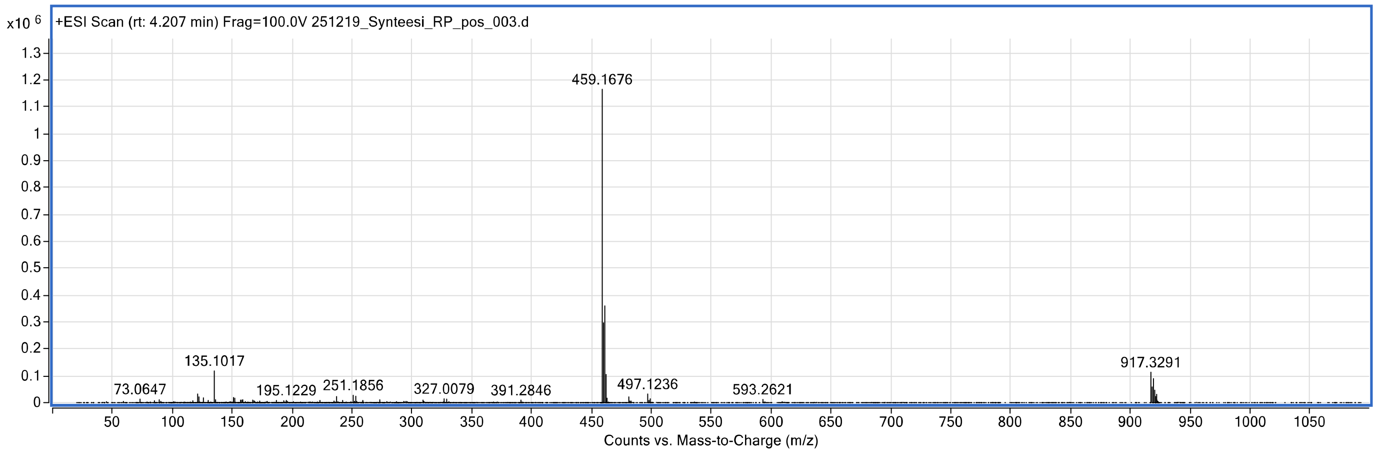

Supplement: Supplementary file 1 — Supplementary Material [file CMDC-21-e202500856-s001.pdf]
